# Supplementary figures and images for: Baseline T cell immune phenotypes predict virologic and disease control upon SARS-CoV infection in Collaborative Cross mice
Source: PLoS Pathog. 2021 Jan 29;17(1):e1009287. doi: 10.1371/journal.ppat.1009287 (PMC7875398; doi:10.1371/journal.ppat.1009287)

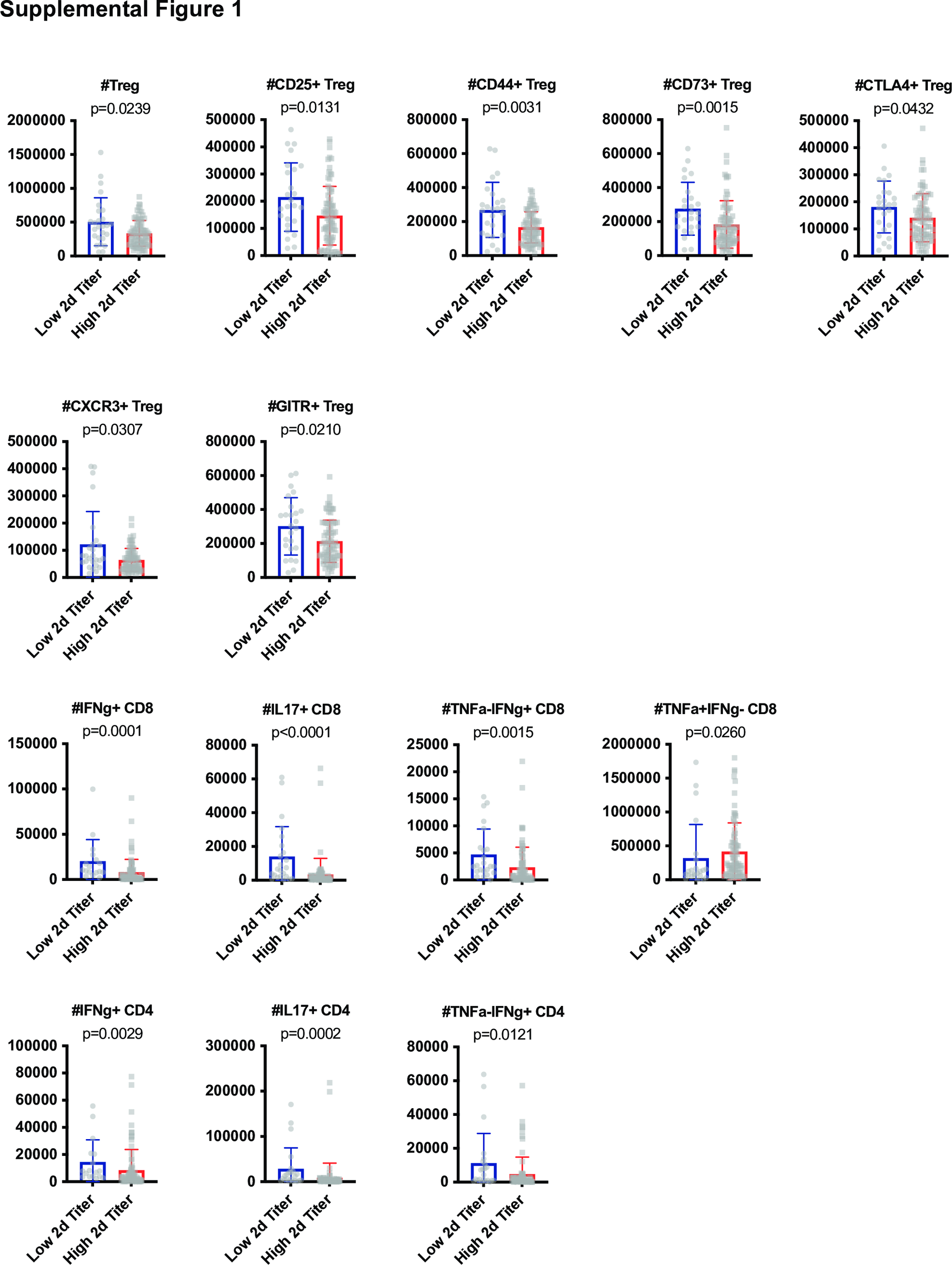

Supplement: S1 Fig — Age-matched female CC-RIX were infected intranasally with SARS-CoV MA15 and lung viral loads at day 2 post-infection were used to select CC-RIX lines with extreme phenotypes: “Low 2d Titer” or “High 2d Titer”, as indicated in Fig 1. Mice from a second cohort of 3–6 age-matched male mice of these selected lines were euthanized and splenic cells analyzed by flow cytometry staining to determine the number of T cells with the indicated phenotype. Statistical significance was determined by Mann-Whitney test. Comparisons are shown for which p<0.05 without adjustment for multiple comparisons. (TIF) [file ppat.1009287.s001.tif]

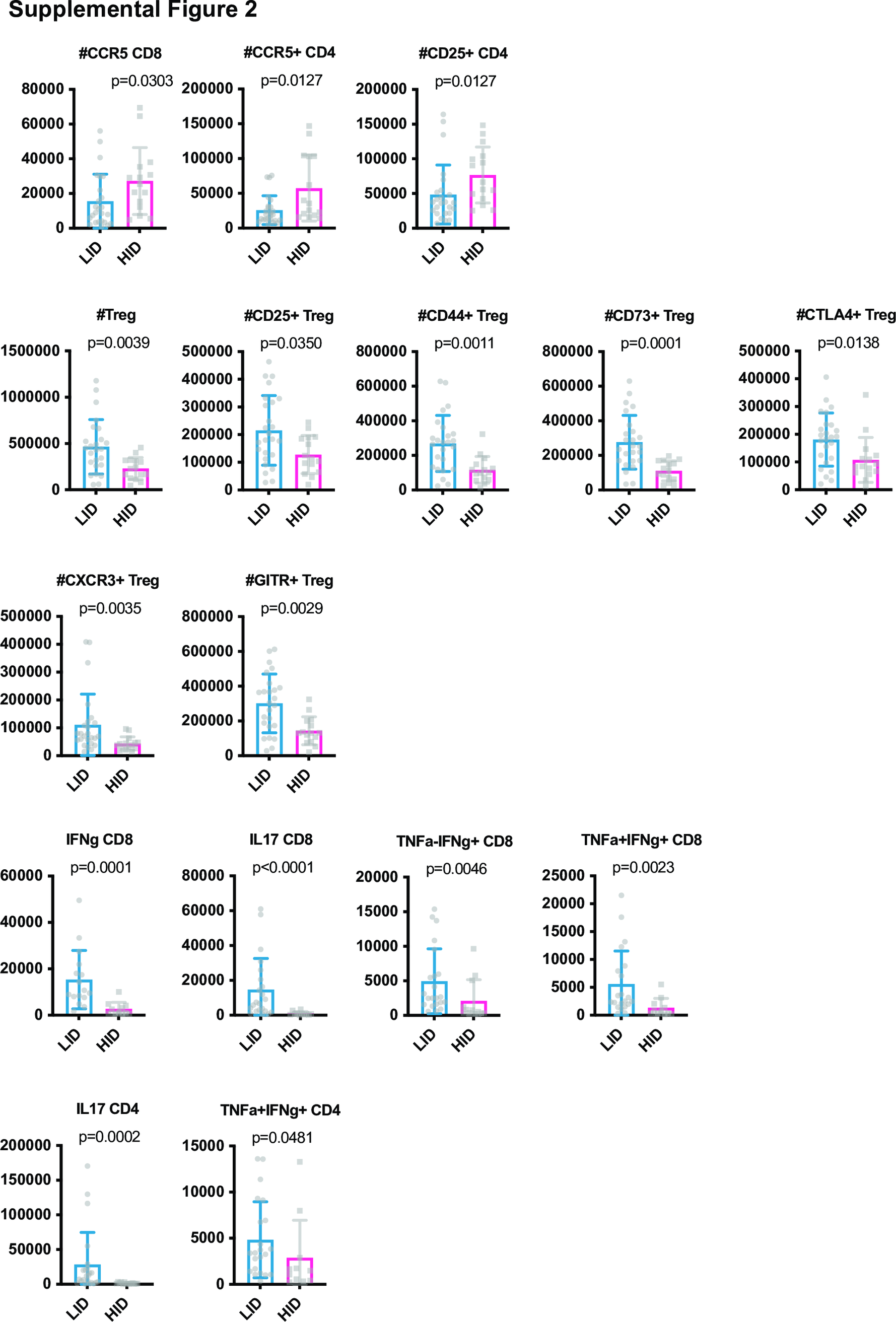

Supplement: S2 Fig — Age-matched female CC-RIX were infected intranasally with SARS-CoV MA15 and mice were monitored for death, weight loss, and lung viral loads. To identify possible baseline immune predictors of both viral replication as well as disease upon infection, we classified CC-RIX lines with extreme phenotypes based on both lung viral loads at days 2 and 4 post-infection, as well as weight loss and mortality. Lines were categorized as “low infection and disease” (LID), which had 0–5% weight loss upon infection, no death, day 2 average lung viral titers of <105 and average day 4 lung viral titers of <104 (N = 5 lines). Conversely, N = 4 lines were categorized as “high infection and disease” (HID) if they experienced greater than 15% weight loss and death, as well as average lung viral titers at day 2 post-infection of >106 and average lung viral titers at day 4 post-infection of >105. Mice from a second cohort of 3–6 age-matched male mice of these selected 9 lines were euthanized and splenic cells analyzed by flow cytometry staining to determine the number of T cells with the indicated phenotypes. Statistical significance was determined by Mann-Whitney test. Comparisons are shown for which p<0.05 without adjustment for multiple comparisons. (TIF) [file ppat.1009287.s002.tif]

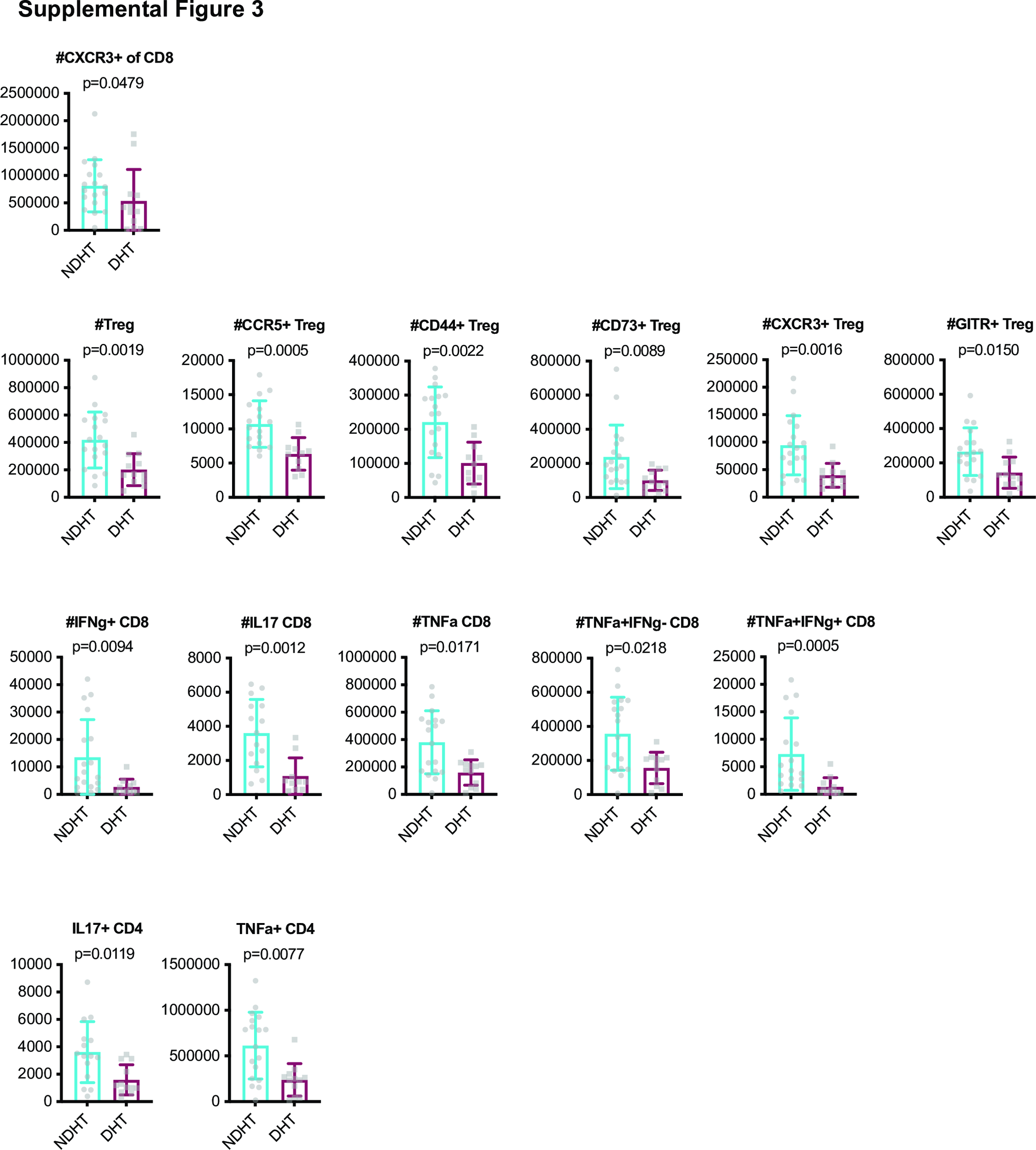

Supplement: S3 Fig — Age-matched female CC-RIX were infected intranasally with SARS-CoV MA15 and mice were monitored for death, weight loss, and lung viral loads. To identify possible baseline immune predictors of disease upon infection with a high early lung viral load, we classified CC-RIX lines with extreme phenotypes based on both lung viral loads at days 2 and 4 post-infection, as well as weight loss and mortality. Lines were categorized as “no disease high titer” (NDHT), which had 0–5% weight loss upon infection and no death despite day 2 average lung viral titers of >107 and average day 4 lung viral titers of >105 (N = 3 lines) and “disease high titer” (DHT; N = 3 lines) if they experienced greater than 15% weight loss and death, as well as average lung viral titers at day 2 post-infection of >107 and average lung viral titers at day 4 post-infection of >105. Mice from a second cohort of 3–6 age-matched male mice of these selected 6 lines were euthanized and splenic cells analyzed by flow cytometry staining to determine the number of T cells with the indicated phenotypes. Statistical significance was determined by Mann-Whitney test. Comparisons are shown for which p<0.05 without adjustment for multiple comparisons. (TIF) [file ppat.1009287.s003.tif]

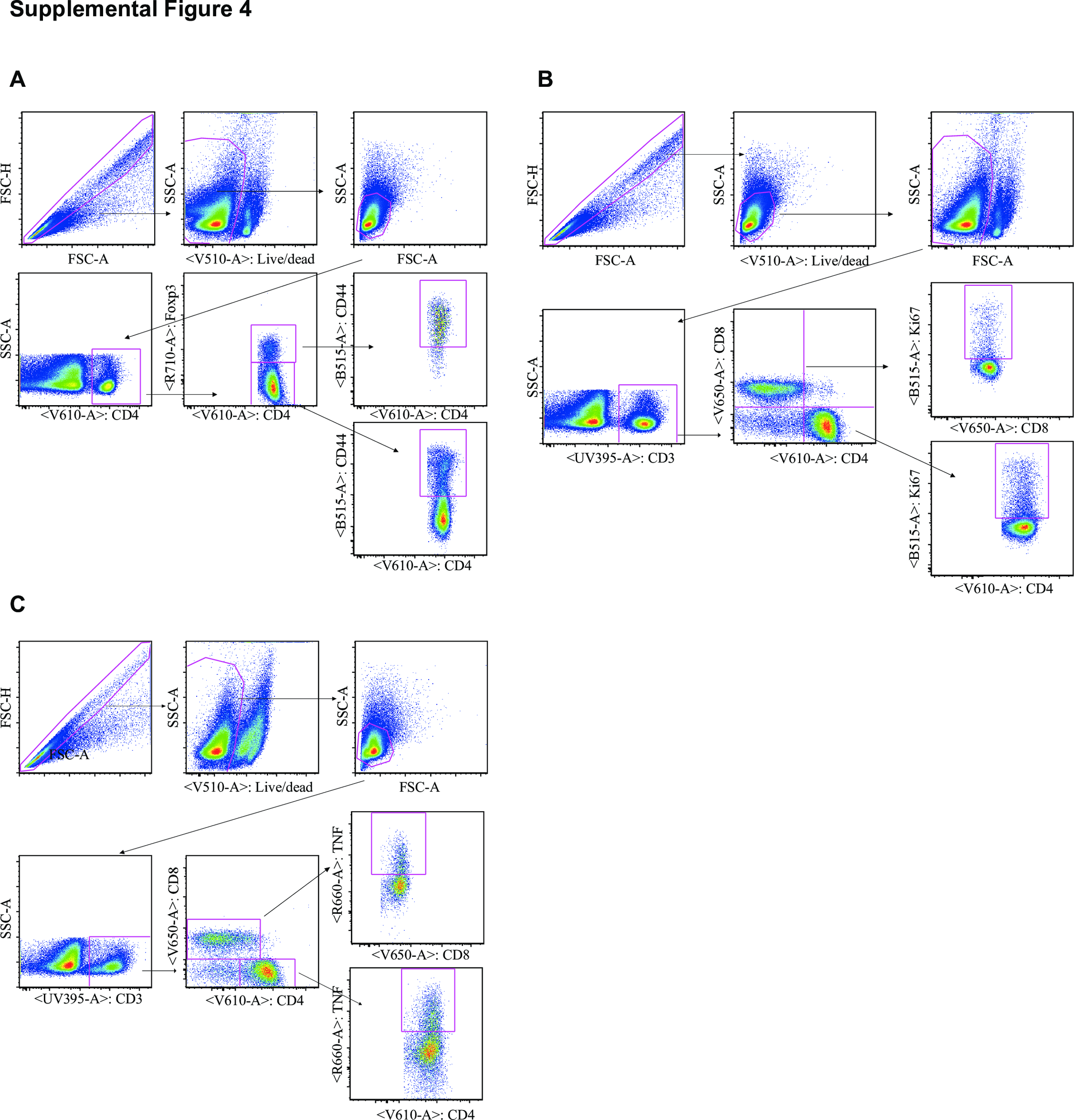

Supplement: S4 Fig — (A) Regulatory T cell panel. The panel is gated in the following order: singlets, live, lymphocytes, CD4+, CD4+ Foxp3-, and CD4+ Foxp3+ (Tregs), followed by activation markers on CD4+ Foxp3- and Tregs. Shown here is CD44, and CCR5, CC25, CD73, CTLA-4, CXCR3, GITR, or ICOS+ cells were also identified for CD4+ Foxp3- and Treg populations. B) T cell panel. The panel is gated in the following order: singlets, live, lymphocytes, CD3+, CD4+ CD8- and CD8+ CD4- T cells, followed by activation markers on CD8+ or CD4+ T cells. Shown here is Ki67, and CCR5, CC25, CD44, CXCR3, or ICOS+ cells were also identified for CD8+ or CD4+ populations. C) Intracellular cytokine staining cell panel. The panel is gated in the following order: singlets, live, lymphocytes, CD3+, CD4+ CD8- and CD8+ CD4- T cells, followed by intracellular cytokines produced by CD8+ or CD4+ T cells. Shown here is TNF, and IFNg and IL-17+ cells were also identified for CD8+ or CD4+ populations (following a 5hr stimulation with αCD3/CD28). (TIF) [file ppat.1009287.s004.tif]
