# Supplementary material for: Baseline T cell immune phenotypes predict virologic and disease control upon SARS-CoV infection in Collaborative Cross mice
Source: PLoS Pathog. 2021 Jan 29;17(1):e1009287. doi: 10.1371/journal.ppat.1009287 (PMC7875398; doi:10.1371/journal.ppat.1009287)
Supplement: S1 Table — RIX lines used in the study, along with d2 and d4 viral loads, are displayed in the table, along with their group designation. Lines with an average lung viral load of less than 105 at day 2 post-infection (N = 8) were considered to be “low titer”, and lines with an average lung viral load of greater than 107 at day 2 post-infection (N = 24) were considered to be “high titer” for further analysis. (DOCX) [file ppat.1009287.s005.docx]

| **S_1Table. CC F1 lines in infection and disease categories** | | | |
| --- | --- | --- | --- |
| **RIX Line** | **d2 Viral Load** | **D4 Viral Load** | **Group** |
| CC042xCC019 | 0 | 847 | Low titer |
| CC030xCC061 | 33 | 333 | Low titer |
| CC018xCC065 | 683 | N/A | Low titer |
| CC030xCC023 | 700 | 0 | Low titer |
| CC052xCC014 | 6333 | 233 | Low titer |
| CC012xCC032 | 46333 | 233 | Low titer |
| CC032xCC013 | 46667 | 67 | Low titer |
| CC011xCC032 | 91500 | 0 | Low titer |
| CC019xCC004 | 10200000 | 27000 | High titer |
| CC068xCC043 | 10350000 | 105750 | High titer |
| CC006xCC039 | 10366667 | 211000 | High titer |
| CC057xCC052 | 11193333 | 28000 | High titer |
| CC062xCC046 | 11333333 | 114667 | High titer |
| CC029xCC071 | 12166667 | 577500 | High titer |
| CC060xCC037 | 13200000 | 14470 | High titer |
| CC041xCC016 | 14100000 | 120700 | High titer |
| CC028xCC024 | 14650000 | 2966667 | High titer |
| CC065xCC010 | 14683333 | 330667 | High titer |
| CC001xCC055 | 14800000 | N/A | High titer |
| CC013xCC041 | 15366667 | 172500 | High titer |
| CC074xCC058 | 16800000 | 128667 | High titer |
| CC004xCC011 | 16816667 | 1003333 | High titer |
| CC026xCC034 | 17500000 | 175667 | High titer |
| CC061xCC025 | 18200000 | 192667 | High titer |
| CC016xCC038 | 18666667 | 116000 | High titer |
| CC056xCC033 | 20333333 | 300033 | High titer |
| CC033xCC046 | 21000000 | 409667 | High titer |
| CC025xCC028 | 23166667 | 640333 | High titer |
| CC042xCC025 | 23666667 | N/A | High titer |
| CC015xCC059 | 26000000 | 80000 | High titer |
| CC055xCC006 | 37333367 | N/A | High titer |
| CC055xCC028 | 47566667 | 33 | High titer |
| CC018xCC065 | 683 | 8400 | LID |
| CC032xCC013 | 46667 | 67 | LID |
| CC030xCC023 | 700 | 0 | LID |
| CC030xCC023 | 33 | 333 | LID |
| CC052xCC014 | 6333 | 233 | LID |
| CC001xCC074 | 3686667 | 688833 | HID |
| CC074xCC058 | 16800000 | 128667 | HID |
| CC025xCC028 | 23166667 | 640333 | HID |
| CC061xCC025 | 18200000 | 192667 | HID |
| CC013xCC041 | 15366667 | 172500 | NDHT |
| CC016xCC038 | 18666667 | 116000 | NDHT |
| CC033xCC046 | 21000000 | 409667 | NDHT |
| CC074xCC058 | 16800000 | 128667 | DHT |
| CC025xCC028 | 23166667 | 640333 | DHT |
| CC061xCC025 | 18200000 | 192667 | DHT |
