# Supplementary material for: Baseline T cell immune phenotypes predict virologic and disease control upon SARS-CoV infection in Collaborative Cross mice
Source: PLoS Pathog. 2021 Jan 29;17(1):e1009287. doi: 10.1371/journal.ppat.1009287 (PMC7875398; doi:10.1371/journal.ppat.1009287)
Supplement: S2 Table — (DOCX) [file ppat.1009287.s006.docx]

| S2 Table. Antibody fluorochromes and clones used in flow cytometry panels | | |
| --- | --- | --- |
| Antibody | **Fluorochrome** | **Clone** |
| CCR5 (CD195) | PE | HM-CCR5 |
| CD3e | Purified | 145-2C11 |
| CD3e | PE-CF594 | 145-2C11 |
| CD3e | BUV395 | 145-2C11 |
| CD4 | BV605 | RM4-5 |
| CD8a | BV650 | 53-6.7 |
| CD25 | APC-eFluor 780 | PC61.5 |
| CD25 | BV650 | PC61 |
| CD28 | Purified | 37.51 |
| CD44 | APC Cy7 | IM7 |
| CD44 | FITC | IM7 |
| CD73 | Pacific Blue | TY/11.8 |
| CD127 | PE-Cyanine5 | A7R34 |
| CTLA-4 (CD 152) | APC | UC10-4B9 |
| CXCR3 (CD183) | PerCP-Cyanine5.5 | CXCR3-173 |
| Foxp3 | Alexa Fluor 700 | FJK-16s |
| GITR (CD357) | PE-Cyanine7 | DTA-1 |
| ICOS (CD278) | PE-Cyanine5 | 7E.17G9 |
| IFN-𝛾 | PerCP-Cyanine5.5 | XMG1.2 |
| IL-17a | FITC | TC11-18H10.1 |
| Ki-67 | FITC | SolA15 |
| Live/dead | Aqua - 405 nm excitation |  |
| TNF | APC | MP6-XT22 |
