# Supplementary material for: Baseline T cell immune phenotypes predict virologic and disease control upon SARS-CoV infection in Collaborative Cross mice
Source: PLoS Pathog. 2021 Jan 29;17(1):e1009287. doi: 10.1371/journal.ppat.1009287 (PMC7875398; doi:10.1371/journal.ppat.1009287)
Supplement: Attachment — Submitted filename: Lund_pointbypointresponse.docx [file ppat.1009287.s007.docx]

December 14, 2020

Dr. Kanta Subbarao

Section Editor

PLOS Pathogens

Dear Dr. Subbarao:

We thank you and our Reviewers for the rigorous and thoughtful review of our manuscript, “Baseline T cell immune phenotypes predict virologic and disease control upon SARS-CoV infection”. There were several concerns raised that we believe we have addressed, and further, we believe that these additional analyses and discussion points greatly strengthened our manuscript. We appreciate the invitation to resubmit this significantly-revised revision that takes into account the reviewers comments, and as suggested in your letter, we have prepared a point-by-point response that outlines how we have addressed each of the reviewer concerns. We thank both you and the Reviewers for this opportunity, as these changes have enhanced our manuscript, and we are grateful for this round of peer-review.

We thank the Reviewers for their assessments that *“The manuscript is generally well-written and the experiments are well-conceived and performed. The reported findings have broad relevance to understanding natural immunity to pandemic betacoronaviruses and point to specific factors that can be further explored in human populations”,* and that *“[the study] would be of interest to the readership of PLOS Pathogens”*. We recognize that our manuscript had shortcomings that we address below, including additional data for clarity and substantially revised text based on suggestions from the reviewers, and we believe our manuscript is significantly strengthened based on these changes.

Below we offer our point-by-point responses to the three reviews of our manuscript. We appreciate your thoughtful consideration, and sincerely hope that you find our work appropriate for publication in *PLOS Pathogens*.

***Part II***

***Reviewer 1:***

*1) The intracellular cytokine staining assay analysis strategy utilized in this manuscript is not described in the methods and appears to have inconsistent or missing data. For example, in Figure 4P, the percentage of positive TNF-IFNg+ CD8 T cells and TNF+IFNg+ CD8 T cells (0.8% and 0.6%, respectively) does not equal the reported percentage of IFNg+ CD8 T cells (3.4%). Many of the other intracellular cytokine staining experiments also have similar inconsistencies. This leads the reader to consider the conclusion that the gates are inconsistently applied across experiments or that additional missing data is collected, but not presented – such as an unreported large population of IL-17+TNF-IFNg+ cells. Depending upon the design of the experiment, which is not clearly reported in this manuscript, SPICE software (available from the NIH) or other visualization programs may provide more consistent visualization that will allow the reader to more clearly identify differences between the analyzed groups. Please address the current inconsistencies (0.8% + 0.6% does not equal 3.4% and others) by providing additional details of the methods employed and consider alternative analysis and visualization strategies to comprehensively present the data you actually collected rather than the few analyses that you selected to perform on the collected data. If an alternative visualization strategy is not employed in the resubmission, then your rationale for not reporting all of the collected data must be stated and gating strategies must be completely demonstrated in supplemental material so that the reader can understand the analysis you chose to perform.*

We thank the reviewer for pointing out that the specific flow cytometry gating strategy should be included in our supplemental figures, as this will aid in understanding where the cell subsets are decended from. To best allow for identification of rare cell populations in mice of varying genetic backgrounds, each cytokine or activation marker is gated on bulk CD4 or CD8 T cells. For example, the gate “% CD4+ IFNγ+” identifies the frequency of CD4+ cells that are IFNγ+ (regardless of expression of any other cytokine). Each of these cytokines or activation markers receives its own gate, and additionally, we also identified cells from the CD4 or CD8 gates (using quadrant gating) that were either IFNγ+ TNFa+, IFNγ+ TNFa-, and IFNγ- TNFa+, as we have previously reported on T cell coexpression of these cytokines in WNV infection. As a result, it is possible that the discrepancy in percentages seen within each category does result from unidentified subsets, as suggested by the reviewer. We included the specific IFNg and TNF analyses because we had specific hypotheses to address (ie that TNF and IFNg are involved in inflammatory and protective immune responses to virus infection, respectively), whereas inclusion of additional and exploratory subsets at this point would introduce additional comparisons to correct for, thereby lowering our statistical power. We have added the gating trees from our FlowJo analysis to the supplemental figures (Supplemental Figure 4) for additional clarity.

***Reviewer #2****:*

*1) The work appears to be entirely a re-analysis of previously published data in reference 28, where in the earlier work, the results from three separate viruses were combined, while here the SARS-CoV-1 results were separated. Has any new data been generated? If not this should be made clear*

In our previously published manuscripts, we identified immune predictors of mortality to RNA virus infection by examining baseline T cell signatures that associated with protection from mortality after infection from flu, SARS-CoV, and WNV infection. In this analysis, rather than binning solely by mortality, we examined CC lines with different immune outcomes to SARS-CoV, such as low or high d2 viral titer, or low infection/disease and high infection/disease (based on weight loss and mortality), to then examine what differences in baseline T cell populations and immune subsets correlated with these outcomes. All data was generated during the CC-RIX screen, and the SARS-CoV data and baseline, pre-infection immune phenotyping data were used in this study. *2) The statistical analysis makes no mention of whether corrections were made for multiple comparisons. Numerous measures are reported and tested, and many more that are not detailed were apparently tested and not reported because they were not significant. Depending on how large these numbers are, the results presented may be much less statistically robust. Several of the observed features are likely themselves correlated (i.e. making one cytokine means a CD8 T cell is also more likely to make 2 cytokines)--why not use some clustering approaches to improve power and reduce multiple testing? This would also identify hidden associations in the data, which, as currently presented, report most findings as largely independent outside of the few correlations shown in figure 3.*

We thank the reviewer for pointing out the need to correct for multiple comparisons. We have now corrected for multiple comparisons using Bonferroni correction, which is a very conservative correction method. Thus, for this adjusted significance cut-off, we used the equation α/n, where alpha was set at 0.05 and N=32 (number of tests performed). We therefore now report this adjusted p-value = 0.05/32 or 0.0015625. Given that we consider this to be a conservative statistical approach, we do discuss findings that meet the unadjusted significance cutoff (of p<0.05), which we also report in the figures. However, for heatmaps, only the p-values corrected for multiple comparisons are reported with an asterisk, and we have added discussion around this analysis to the text.

In addition, the reviewer is correct that many measures are likely to be related (for example, Treg frequency and T cell activation markers), though it is difficult to determine which measures are directly related without significant additional experimentation (for example, reducing the number of Tregs pre-infection, and then testing for the effect on all other immune phenotype plus disease phenotype upon SARS-CoV infection). *3) The authors discuss the possibility that the CD8+ CD44+ cells might be "virtual memory" T cells or bona fide memory T cells but apparently did not originally collect the data to distinguish these possibilities. Similarly they did very little characterization of the Treg populations, which can be divided various ways (induced, thymic etc). Given that these are pre-infection profiles, couldn't these be generated quite easily by looking in additional mice? It would strengthen the paper significantly.*

We agree with the reviewer that in retrospect, we wish our screening panel had contained additional markers such as CD49d and CD122 in order to distinguish virtual memory T cells, and that we had additional markers included to better characterize Treg origin. The CC-RIX crosses in the screen were specifically generated for this study, so even the prospect of remaking a subset of these mice for a smaller screen would be a large undertaking of resources and would take a considerable amount of time. Furthermore, given reduced laboratory operations due to the pandemic, we estimate that it would take more than 1 year to complete such additional studies, and thus we do not include them in this revision. We hope that the large dataset presented and analyzed herein will be considered to be of sufficient interest without this additional data, and we propose that this data will be useful as others (beyond our group) consider follow-up studies using the CC.

***Reviewer #3:***

*1) I think it is imperative to include gating strategies for the FACS analysis and to also include representative facs plots to show raw data. This is particularly important for phenotypes/markers and the ICS. This can be in the main figures or as supps.*

We agree that a supplemental figure of gating strategies for each panel, as well as representative FACS plots of the gating strategy, can help clarify the origins of the subgroups identified in our manuscript. Both have been added to the supplemental figures (Supplemental Figure 4). *2) Are absolute numbers available to be include in the analyses?*

Yes, absolute numbers are available for all flow cytometry data included in this study. We have added matched absolute number data for frequency data shown in Figures 2-5 to the supplemental figures (Supplemental Figures 1-3). *3) The weight loss data should be shown in some way. Potentially as the viral titres in Fig1A would be informative for max weight loss or area under the curve. Alternatively or additionally, the weight loss curved across time can be shown for the selected CC-RIX lines in figs 4 and 5.*

We thank the reviewer for pointing out that the weight loss data should be shown, as this adds visual clarity to the clinical data classifications we also use, such as LID vs HID. Weight loss for each of the CC-RIX lines screened has been added to figure 1, as well as for the group comparisons in figures 4 and 5.

***Part III – Minor Issues: Editorial and Data Presentation Modifications*** *Please use this section for editorial suggestions as well as relatively minor modifications of existing data that would enhance clarity.*

***Reviewer #1:***

*1) The description of the experiment in the first section of the Results – lines 131-135, while comprehensive, is difficult to follow. An additional figure, or a Figure 1A, outlining the experimental design would be of assistance to expedite the reader’s ability to grasp the overall experimental design. Especially the relationship between the main survival/weight loss study and the lung viral load measurements on separate cohorts of mice. Unclear how many mice per group were used from how many CC-RIX lines in the lung viral load portion (Figure 1 A and B).*

We agree with the reviewer that an additional figure outlining the experimental design would aid in clarity, and be visually more clear to the reader. In accordance with this excellent suggestion, we have included this information in Figure 1A. We have also added additional details in the text and in each graph to clarify the number of mice included in each of the analyses. *2) You variably refer to the CC-RIX mouse lines with high viral titers at day 2 (N=24) and those with low viral titers at day 2 (N=8) as “mice with superior virologic containment at day 2 post-infection” (line 152) or “lines with low viral titers at day 2 post-infection” (line 156). Please be consistent throughout the manuscript in the terminology that you use to refer to each group of mice – the high titer group and the low titer group. Furthermore, the LID/HID and NDHT/DHT groups are also variably referred to; therefore I would suggest introducing each group with an abbreviation and then abbreviating the groups moving forward from that point.*

We again thank the reviewer for pointing out that using the abbreviation for our groups consistently would aid in understanding of the experimental comparisons. We have encorporated this suggestion by abbreviating the groups consistently within the text after initial introduction (please see marked up manuscript file). *3) Please include the number of analyzed biologic replicates (N) on the graph itself for each column in each grouped experiment you performed (Figure 2 A-F, Figure 3 A-G, Figure 4 A-M, Figure 5 A-G). Please state the minimum number of mice from each of the studied groups in the figure legends (e.g. How many mice from each of the 5 individual LID lines are presented in figure 4B vs 4L? Was this at least 3 mice from each line?).*

We thank the reviewer for this suggestion to aid in the reader’s understanding of the data by labeling the number of biologic replicates on the figure legends, as well as on the graphs themselves. 3-6 mice per line were used in each cohort, and our graphs and legends have been updated to reflect this. *4) Please be consistent with your terminology for the LID and HID groups – there is variable terminology and then unintroduced abbreviations in the section starting at line 193.*

As with point #2, we agree that consistent use of our abbreviated groups would aid in the reader’s understanding of the data displayed in each graph. We have updated the text accordingly. *5) Figure 4 N is not referred to in the main text (lines 221 and 223 do not mention it).*

We thank the reviewer for identifying this labeling error- we have updated the text to include Figure 4N. *6) Line 243 states you are looking at CD25+ and CCR5+ CD4 T cells, however Figure 5D is labeled %CCR5 of CD8.*

The reviewer is correct that the text did not correspond with the figure label and legend. We have corrected this error within the text*.

7) I question the connection you make in the first paragraph of the discussion between understanding individual pre-infection risk of severe disease in people living in the community and the allocation of PPE/treatments/vaccines. Mitigating spread among all members of the community requires PPE, specifically masking, therefore an immunologic assay assessing T cell function of a human patient before distribution of PPE to that individual seems at best more burdensome than just manufacturing more PPE and certainly does not help to prevent community spread. Furthermore, the translation of your work to human subjects – even if it does translate from mouse adapted SARS-CoV to SARS-CoV-2 – is a distant reality, not something that can be rapidly applied at this time before the completion of initial vaccine and treatment efforts for SARS-CoV-2. I suggest you consider rewording the introduction to your discussion section. The points you make in lines 266-271 are the critically important aspects of your work. The direct applicability to human patients, especially those infected with SARS-CoV-2, is less important at this stage. The quality of the work stands on its own and does not have to be stretched to fit an application to the current pandemic.*

We thank the reviewer for the acknowledgment that the quality of our work can stand alone as we identify baseline, circulating T cell signatures that predict severe infection and disease outcomes upon SARS-CoV infection. We have edited the discussion to focus our attention on this goal. The reviewer is certainly correct that PPE allocation would in no way be altered by our findings, and so we have removed mention of that in the first paragraph of the revised Discussion.

*8) Lines 365-366 – this sentence is not clear.*

We agree with the reviewer that this sentence may only have been clear to us within the context of our experimental design. Two different CC-RIX lines were included which appeared twice in the screen, once measured in the beginning and once towards the end of the five years of this program to specifically assess and control for batch and seasonal effects. We have expanded this sentence in the methods for clarity. *9) Lines 373-376 – were these experiments carried out at both UW and UNC? It appears as though UNC is the facility with the BSL-3 facility. Reading through the groups’ previous manuscript from the Journal of Infectious Diseases earlier this year, the wording is very similar to that used here and the discussion of that manuscript mentions that only the WNV infections occurred at UW, whereas the SARS-CoV infections occurred at UNC. Did the infections in this manuscript occur at both locations?*

We have updated the text to be more clear on where these different expriments were carried out. The reviewer is correct that UNC is the facility with the BSL-3 lab, and all SARS-CoV infections and experiments were carried out there. The baseline flow cytometry data in this study comes from the WNV-arm of the screen, which was conducted at UW. All mock-infected animals were housed at UW for these studies. We have updated the methods to identify which cohorts of mice were used for each type of data generation. *10) Lines 395-398 – I can appreciate that the methods used for flow cytometry in this manuscript have been well-established by this group. This becomes clear reading through the methods and supplemental figures for reference #27. However, since four of the five figures from this manuscript exclusively report flow cytometry data, can you please report in the methods section: 1) the specific antibody clones that you used in this manuscript, 2) the organization of the panels that you stained your samples with in this manuscript (i.e. we used CD3, CD4, CD8, IL-17, IFN-gamma, and TNF-alpha together in our intracellular panel; we used CD3, CD4, CD8, CD62L, CD44 together in a surface panel; etc.), 3) the flow cytometer that acquired the data, 4) the software used to analyze the flow cytometry data.*

We agree with the reviewer that while the specifics of the methods are elaborated on in our previous manuscripts, it would be helpful to the reader to briefly expand the methods with these details specific to this study. We have added a supplemental table with antibody clones, and updated the methods text with the additional details of our staining panels. *11) Lines 400-403 – Did you adjust your p-values for multiple comparisons? It appears that each experiment contains between 12 and 31 comparisons. The false-discovery rate should be appropriately accounted for.*

Yes- p-values have now been adjusted for multiple comparisons using Bonferroni correction (with n=32; please see Reviewer #2, part II, point 2 for additional info).  *12) Lines 423-426 – Please provide additional information regarding panel A – the reader must know how many mice of each CC-RIX line were infected and analyzed in each group – at least a range. (i.e. we infected groups of between ## and ## mice intranasally with SARS-CoV MA15). Furthermore, how many lines of CC-RIX mice are represented in this figure? It appears to be close to the approximate 100 number that you mention in line 132 – however, please state the exact number of CC-RIX lines you analyzed and present in this experiment.*

We thank the reviewer for pointing out these missing details from the figure legends and methods. We have updated the text to clarify that 106 unique CC-RIX lines were used in the screen, with 18-28 mice per RIX used in the SARS-CoV study cohort and 3-6 mock infected mice used for the baseline flow cytometry cohort. *13) In Figure 2 G and H, Figure 3 H, Figure 4 N-P and Figure 5 H-J, please indicate the values with a significant difference in the panels themselves rather than just the legend. Perhaps an asterisk for those values with statistical significance after multiple comparison testing?*

We thank the reviewer for this suggestion, and have updated the figure panels with an asterisk for statistical significance (after adjustment for multiple comparisons) next to those values.

*Reviewer #2: None noted--the paper is very clearly written.*

*Reviewer #3: Given the large number of studies that are currently being published on SARS-CoV-2 in different species I think it is important to: i) include mice or CC in the title and ii) perhaps change SARS-CoV to SARS-CoV-1 in title and throughout the text to avoid confusion especially since COVID-19 is mentioned in abstract and introduction.*

We thank the reviewer for this excellent suggestion. We have updated the title to add “in Collaborative Cross mice”, to thereby make it clear that our studies were carried out in genetically diverse mice. We have left reference to SARS-CoV, as this is the official name for the virus that causes SARS, the syndrome first reported in 2003, whereas SARS-CoV-2 is the virus that causes COVID-19.

Thank you in advance for your consideration and reviews that have resulted in substantial improvements in our manuscript. Please don't hesitate to contact me with any questions or if I can provide additional details.

Sincerely yours,

Jennifer Lund

Associate Professor

Vaccine and Infectious Disease Division

Mail Stop E5-110

T 206.667.2217

F 206.667.7767

jlund@fredhutch.org
